# Editorial: Advances in bronchoscopic techniques for the diagnosis of lung cancer

**DOI:** 10.3389/fonc.2024.1428006

**Published:** 2024-05-21

**Authors:** Martina Ferioli, Micaela Romagnoli, Piero Candoli

**Affiliations:** ^1^ Interventional Pulmonology Unit, IRCCS Azienda Ospedaliero-Universitaria di Bologna, Bologna, Italy; ^2^ Pulmonology Unit, Cà Foncello Hospital, AULSS2 Marca Trevigiana, Treviso, Italy

**Keywords:** interventional pulmonology, bronchoscopy, lung cancer, diagnosis of lung cancer, diagnosis of lung nodule

Interventional pulmonology uses minimally invasive techniques to diagnose and treat lung cancer, pleural disease, and many types of complex airway and lung disorders. Over the past decade, interventional pulmonology has seen significant progress in the field of endoscopic procedures, particularly in the diagnosis, staging, and sometimes treatment of lung cancer. The research topic “*Advances in Bronchoscopic Techniques for the Diagnosis of Lung Cancer*” brings together diverse contributions that showcase the latest innovations in bronchoscopy. Here we highlight the innovative advances presented in the articles within this Research Topic from the perspective of an interventional pulmonologist.

When faced with a lung lesion suspected to be lung cancer on chest computed tomography (CT) it is imperative to evaluate the least invasive and most effective diagnostic approach. Among the potential options, the bronchoscopic approach offers the advantage of directly visualizing the airways and the ability to perform biopsies of mucosal alterations. In their original research article, Zhu et al. employed narrow-band imaging (NBI), an endoscopic technique for evaluating surface patterns and microvascular architecture using a narrow spectrum of light that retains only blue and green wavelengths. The study included 916 patients with clinical or radiologic suspicion of central lung cancer or patients undergoing follow-up after curative lung cancer surgery, all of whom underwent bronchoscopy with NBI. Compared with pathological findings, NBI demonstrated diagnostic sensitivity, specificity, positive predictive value, and negative predictive value of 91.7%, 84.9%, 97.6%, and 61.5%, respectively. The article also describes the predominant vascular pattern in relation to the histologic types.

If the lung lesion is peripheral rather than central, it can still be accessed with a bronchoscope using other technologies, such as fluoroscopy and/or a radial endobronchial ultrasound (R-EBUS) probe. This, inserted through the working channel of a flexible bronchoscope or catheter (guide sheath), facilitates real-time imaging of the surrounding tissue, enabling the clinician to confirm the location of the lesion. Hu et al., in their original research article, analyzed the clinical significance of the resistance encountered by the R-EBUS probe when passing through peripheral lung lesions for diagnostic purposes. Probe resistance was classified as type I (no resistance), type II (moderate), and type III (high). A significant difference in the diagnostic yield between malignant and benign disease was detected in type II (p = 0.008). Additionally, the authors assessed the number of biopsies required to reach a diagnosis in different types of resistance and found that in types II and III, the yield increased to a plateau with serial biopsies up to the fourth one, whereas in type I, significantly limited tissue specimens could be obtained with each biopsy.

In recent years, various methods for locating and sampling peripheral lesions have been developed to enhance the diagnostic yield of bronchoscopy. These include electromagnetic navigation bronchoscopy, virtual bronchoscopy, augmented fluoroscopy, cone beam CT-assisted bronchoscopy, and robotic bronchoscopy. In their brief research report, Lanfranchi et al. described the utilization of virtual bronchoscopy and the bronchoscopic trans-parenchymal approach in nine cases where bronchoscopy with R-EBUS failed to reach the lesion. They achieved a diagnostic yield of 77.8% with a low complication rate.

Once the lesion is reached, regardless of the technology used to locate it, sampling is performed. Rapid On-Site Evaluation (ROSE) allows preparation and staining of the specimen obtained by bronchoscopy during the procedure, facilitating immediate cytopathological evaluation and providing feedback to the operator regarding specimen adequacy and potential diagnosis. Yan et al. presented an original research project that attempted to develop an artificial intelligence (AI) system for ROSE using deep learning techniques. They reported an accuracy of 92.97% on the internal testing dataset and 90.26% on the external testing dataset, achieving a performance in distinguishing benign from malignant lesions comparable to that of a real cytopathologist. This project demonstrates the potential role of ROSE-AI in clinical practice, for which additional validation is obviously needed.

Bronchoscopy procedures could be used not only for diagnosis but also to assist in therapy. The original research by Zeng et al. outlined the use of electromagnetic navigation bronchoscopy to localize and mark multiple ground grass lesions with methylene blue to facilitate video-assisted thoracoscopic surgery (VATS). In total, 57 patients underwent ENB-guided bronchoscopic localization followed by VATS, resulting in the resection of a total of 150 nodules, with an overall malignancy rate of 66%, a localization accuracy of 94%, and a maximum distance between mark and lesion of 8 mm.

Bronchoscopy may also play a definite therapeutic role in selected cases of endobronchial neoplasms. In their case report, Wang and Hou describe a case involving an adolescent patient with a benign leiomyoma that was successfully removed using an endoscopic approach with endoscopic submucosal dissection. The majority of the tumor was excised using an electrocautery snare and cryoprobe, followed by the use of a hybrid knife to dissect the tumor stump and base. Finally, argon plasma coagulation was applied to treat the wound surface.

Taken together, the original articles, the brief research report, and the case reports collected in this Research Topic represent an example of the great innovations that have characterized interventional pulmonology in recent years. These advances are aimed at achieving earlier diagnosis of suspected lung neoplasms through the development of new technologies. Moreover, the expanding landscape of specialized oncologic therapies underscores the importance of obtaining sufficient cytohistologic material for comprehensive analyses, such as immunologic and molecular assessments. Such analyses play a crucial role in guiding the selection of the most appropriate therapeutic approach tailored to both the disease and the individual patient’s needs. Coordination and collaborative updates among the various specialties involved in the treatment of lung cancer will be the cornerstone of cancer management in the future.

## Author contributions

MF: Conceptualization, Writing – original draft, Writing – review & editing. MR: Supervision, Writing – original draft, Writing – review & editing. PC: Supervision, Writing – original draft, Writing – review & editing.

